# PET/MRI of Hepatic 90Y Microsphere Deposition Determines Individual Tumor Response

**DOI:** 10.1007/s00270-015-1285-y

**Published:** 2015-12-31

**Authors:** Kathryn J. Fowler, Nichole M. Maughan, Richard Laforest, Nael E. Saad, Akash Sharma, Jeffrey Olsen, Christina K. Speirs, Parag J. Parikh

**Affiliations:** Department of Radiology, Washington University, Campus Box 8131, 510 S. Kingshighway Blvd, St. Louis, MO 63110 USA; Department of Biomedical Engineering, Washington University, Campus Box 1097, 1 Brookings Dr, St. Louis, MO 63130 USA; Department of Nuclear Medicine, Washington University, Campus Box 8225, 510 S. Kingshighway Blvd, St. Louis, 63110 MO USA; Department of Radiation Oncology, Washington University, Campus Box 8224, 4921 Parkview Place, St. Louis, MO 63110 USA

**Keywords:** Radioembolization, Dosimetry, Imaging, PET, Liver/hepatic, Cancer

## Abstract

**Purpose:**

The purpose of our study is to determine if there is a relationship between dose deposition measured by PET/MRI and individual lesion response to yttrium-90 (^90^Y) microsphere radioembolization.

**Materials and Methods:**

26 patients undergoing lobar treatment with ^90^Y microspheres underwent PET/MRI within 66 h of treatment and had follow-up imaging available. Adequate visualization of tumor was available in 24 patients, and contours were drawn on simultaneously acquired PET/MRI data. Dose volume histograms (DVHs) were extracted from dose maps, which were generated using a voxelized dose kernel. Similar contours to capture dimensional and volumetric change of tumors were drawn on follow-up imaging. Response was analyzed using both RECIST and volumetric RECIST (vRECIST) criteria.

**Results:**

A total of 8 hepatocellular carcinoma (HCC), 4 neuroendocrine tumor (NET), 9 colorectal metastases (CRC) patients, and 3 patients with other metastatic disease met inclusion criteria. Average dose was useful in predicting response between responders and non-responders for all lesion types and for CRC lesions alone using both response criteria (*p* < 0.05). D70 (minimum dose to 70 % of volume) was also useful in predicting response when using vRECIST. No significant trend was seen in the other tumor types. For CRC lesions, an average dose of 29.8 Gy offered 76.9 % sensitivity and 75.9 % specificity for response.

**Conclusions:**

PET/MRI of ^90^Y microsphere distribution showed significantly higher DVH values for responders than non-responders in patients with CRC. DVH analysis of ^90^Y microsphere distribution following treatment may be an important predictor of response and could be used to guide future adaptive therapy trials.

## Introduction

Radioembolization of hepatic malignancies delivers higher radiation dose to tumors than surrounding liver parenchyma [1–6]. This is achieved by selective injection of a high-energy radiation source, ^90^Y [Yttrium-90, 0.93 MeV; tissue penetration mean 3.9 mm, maximum 11 mm], into the hepatic artery supplying the lobe or region of the tumor.

The typical pre-procedure work-up includes diagnostic imaging with contrast-enhanced computed tomography (CT) or magnetic resonance imaging (MRI) to determine tumor burden, angiography to identify anomalies that may lead to non-target embolization, and evaluation for hepatopulmonary shunting via technetium-99m-labeled macroaggregated albumin (^99m^Tc-MAA) injection with SPECT/CT [6, 7]. While generally well tolerated, the main complications of radioembolization are liver toxicity from radiation exposure and non-target embolization [8–15]. Response is generally heterogeneous between patients, even those with the same tumor types [16, 17].

Because of the importance of selective delivery and adequate dose to tumor coverage, there is growing interest in quantitatively and qualitatively imaging ^90^Y microspheres within the liver after delivery. Historically, post-therapy imaging was done with Bremsstrahlung imaging [18, 19]; however, positron emission tomography (PET) has generally replaced SPECT due to the need for higher resolution imaging to localize dose distribution [18–29]. In a recent study focusing on hepatocellular carcinoma patients, Lea et al. demonstrated wide variation in measured tumor and parenchymal doses on PET/CT following lobar administration of glass microspheres [30]. This wide variability may lead to heterogeneous tumor response and the potential to under-dose tumors while over-dosing background liver [27, 31]. The authors suggested the need for continued patient-specific dosimetry methods.

The purpose of our study was to assess the feasibility of PET/MRI to evaluate the ^90^Y microsphere deposition and the resultant dose delivered in individual lesions. The second purpose was to assess whether the measured dose was related to local tumor response. To our knowledge, this is the first series of ^90^Y PET/MRI patients published with clinical follow-up.

## Materials and Methods

### Patient Sample

Between October 1, 2012 and April 17, 2014, patients undergoing radioembolization for any indication were recruited and consented on an IRB-approved protocol (NCT01744054) for PET/MRI imaging on a Siemens Biograph mMR (Siemens Healthcare, Erlangen, Germany). 26 of these patients had imaging follow-up as defined as contrast-enhanced imaging at 3 months or later. Two patients were excluded from analysis due to inability to confidently draw contours around their initial lesion or lesion on follow-up imaging, leaving 24 patients for this analysis. Patient demographics, treatment details and tumor characteristics are listed in Table 1. All patients underwent ^90^Y microsphere delivery pretreatment evaluation and delivery according to standard procedures. Two patients received whole liver treatment as opposed to standard lobar treatment to prevent further delay of chemotherapy.

**Table 1:** Patient demographics and treatment information

Patient demographics						
Tumor type	Age and gender	#Tumors correspond to follow-up	Total tumor volume(cc)	Delivery site (glass or resin microspheres)	Delivered activity (GBq)	Contrast agent for PET/MRI
HCC						
	83, F	1	61.78	Left lobe (resin)	0.7	Eovist
	83, F	1	5.0	Right lobe(resin)	1.03	Eovist
	75, F	1	1514.3	Left lobe (glass)	2.99	Eovist
	61, M	1	157.7	Whole liver (glass)	3.94	Eovist
	77, M	1	185.0	Left lobe (glass)	2.21	Eovist
	62, M	1	549.0	Left lobe (glass)	1.09	Eovist
	74, M	3	376.7	Right lobe (glass)	4.96	Multihance
	73, F	1	27.7	Left lobe (glass)	0.82	Multihance
NET						
	52, M	9	623.6	Right lobe (glass)	2.2	Eovist
	40, M	6	21.0	Right lobe (glass)	0.4	Eovist
	75, M	2	494.6	Left lobe (resin)	0.9	Multihance
	48, F	8	27.7	Right lobe (resin)	0.7	Multihance
CRC						
	52, M	1	257.8	Right lobe (resin)	1.6	Eovist
	59, M	2	2393.3	Right lobe (resin)	1.4	Eovist
	57, M	3	212.1	Right lobe (resin)	0.9	Eovist
	82, F	2	73.0	Right lobe (resin)	1.0	Eovist
	68, M	4	100.2	Whole liver (resin)	3.2	Eovist
	60, F	10	223.2	Right lobe (resin)	1.0	Multihance
	53, M	3	40.9	Right lobe (resin)	1.6	Multihance
	48, M	12	1681.2	Right lobe (resin)	1.5	Multihance
	54, M	5	356.7	Right lobe (resin)	2.0	Multihance
Esophageal						
	63, M	3	326.4	Right lobe (resin)	1.6	Multihance
Breast						
	57, F	3	39.1	Right lobe (resin)	1.0	Multihance
Thymic carcinoid						
	49, M	4	529.1	Left lobe (resin)	0.9	Multihance

Current methods for prescribing radioembolization dose, as recommended by the manufacturer [6], differ in part by the particle type (resin versus glass). Glass microsphere (TheraSpheres, BTG International, Canada) dose prescription is determined by the following equation:

Infused liver volume (independent of tumor burden)

$${\text{A }}\left( {\text{GBq}} \right) = \left[ {{\text{D}}_{\text{desired}} \left( {\text{Gy}} \right) \times {\text{M}}_{\text{target liver}} \left( {\text{kg}} \right)} \right]/ 50$$

These microspheres are typically delivered to patients with unresectable hepatocellular carcinoma (HCC) and occasionally metastatic neuroendocrine tumors (NET). Resin microspheres (SIR-spheres, Sirtex Medical Ltd., Sydney, Australia) may be administered by body surface area method:

BSA and  % tumor burden

$${\text{A }}\left( {\text{GBq}} \right) = {\text{BSA }}{-} \, 0. 2 + \left( {\% {\text{ tumor involvement}}/ 100} \right).$$

These microspheres are typically delivered to metastatic lesions in the liver, such as colorectal cancer (CRC) and NET. These methods also require estimation of a lung shunt fraction prior to treatment with reduction in dose if the lung shunt fraction is above 10–20 %. The average activity delivered to patients was 1.65 GBq (range: 0.4–4.96 GBq), which correlates to a dose of 120–130 Gy in the treated lobe of the liver. An inherent limitation of the current strategies for estimating dose is the assumption of uniform delivery within the segment, section, or lobe to which radioactivity is delivered.

### Post-treatment ^90^Y PET/MRI Acquisition Parameters

Post-procedural PET/MRI consisted of routine liver sequences (detailed below) and simultaneous PET data acquisition. The PET component consists of 8 rings of 56 detector blocks, each with a 4 × 4 × 20 mm LSO (lutetium oxyorthosilicate) crystals with scintillation light readout using avalanche photodiodes. The coincidence window time resolution is 5.86 ns. The spatial resolution is 4.3 mm (reconstructed resolution closer to 6 mm) at FWHM. Imaging was done within 66 h (range 0.75–66 h) of ^90^Y radioembolization based on patient and scanner convenience.

Patients were positioned with arms raised, and 20–40 min of PET data were acquired in a single station to cover the liver and lower thorax. The MR sequences used were a 2-point DIXON for attenuation correction, T2 Turbo spin-echo (TSE) fat-suppressed axial respiratory navigated, in/opposed-phase dual-echo gradient recall T1-weighted, pre-contrast volumetric interpolated breath hold examination (VIBE), dynamic post-contrast VIBE, coronal post-contrast VIBE, diffusion-weighted images (*b* values 50, 400, 800), axial non-fat-suppressed T2-weighted, radial free-breathing VIBE, and a 20-min delayed VIBE in the axial and coronal planes (for gadoxetic acid enhanced MRI only). Intravenous contrast consisted of gadoxetic acid (Bayer Pharmaceuticals; dose of 0.05 mmol/kg) administered at 1 ml/second or gadobenate dimeglumine (Multihance, Bracco Diagnostics; dose of 0.1 mmol/kg) administered at 2 ml/second.

### ^90^Y PET/MR Reconstruction Parameters

 Tomographic images were generated by iterative reconstruction [3D-Ordered Subset Expectation Maximization (OSEM)] using the following parameters for the Siemens Biograph mMR: 3 iterations, 21 subsets, 172 × 172 matrix, post-processing Gaussian filter of 5 mm in full width at half maximum, and with point spread function compensation, resulting in a voxel size of 4.17 × 4.17 × 2.02 mm. The parameters for reconstruction were based upon phantom studies conducted at our institution to determine the optimal recovery coefficient with a moderate noise level over a wide range of activity levels [33]. Attenuation correction was derived from the 2-point DIXON MR VIBE sequence (TR = 3.6 ms, TE1 = 2.46 ms and TE2 = 1.23 ms, flip angle of 10°). Scatter correction was applied using a single scatter simulation technique as provided by the manufacturer. The attenuation of the PET caused by the bed and fixed MRI coils was automatically integrated into the attenuation maps. The scanner was calibrated for absolute activity concentration using a 20 cm diameter ^68^Ge cylinder containing a known activity concentration and cross-calibrated to the laboratory dose calibrator with a similarly configured ^18^F-filled cylinder. Since ^90^Y was not a listed nuclide for PET acquisition on the Siemens Biograph mMR scanner, we used the settings of ^86^Y for data acquisition and image reconstruction. The scanner calibration factor (ECF) used a ratio of the positron fractions between the selected isotope for scanning (^86^Y) and ^68^Ge, and then we manually corrected for ^90^Y by scaling the reconstructed image intensity by the relative β + decay branching ratios and decay constants of ^86^Y and ^90^Y. Our previous phantom study with ^90^Y chloride solution showed that the calibration from ^68^Ge was accurate [33].

### Image Evaluation and Post-processing

PET and MRI data were reviewed on MimVista (MIM Software, Cleveland, OH) by a board-certified, fellowship-trained MRI radiologist (10 years of experience in abdominal imaging), using rigid registration to align and fuse the liver boundaries. MR sequences were co-registered, and tumor contours, lobar, and whole liver contours were drawn primarily on the Gadoxetic hepatobiliary phase images (20 min delay) or on arterial or portal venous images for patients who received an alternate contrast agent. Images were assessed qualitatively for expected distribution of dose based on injection site and extrahepatic deposition. Regions of interest were drawn over the paraspinal muscles to derive a background value. Dose maps were calculated by convolution of the activity concentration images from ^90^Y PET images and a voxelized radiation dose kernel [34]. In short, images were re-sampled on 3-mm cubic voxels, convolved with MIRD-17 3D 3 mm voxel dose-point kernel, and finally re-sampled on the original voxel size, similar to Lea et al. [30]. Image processing was performed using an application written in MATLAB R2012a (Mathworks, Natick, MA). Voxel residence times were calculated using immediate uptake and physical decay only. Based upon the PET-generated dose maps, dose volume histograms (DVH), which plot the minimum dose (Gy) to a given volume (%) of a specified region of interest, were generated for each lesion measuring ≥1 cm diameter for RECIST criteria and ≥1 cc for vRECIST criteria. Smaller lesions were not analyzed due to inability to confidently draw contours and identify the lesions on follow-up imaging. To determine treatment response, follow-up imaging was acquired on all patients according to standard of care intervals. Contours were drawn around the same lesions as contoured on the initial imaging time point (with initial and follow-up imaging assessed in the same session to allow accurate matching). Standard RECIST criteria were used for differentiating responders (≥30 % decrease in the longest tumor diameter), non-responders (≥20 % increase in the longest tumor diameter), and stable lesions (else) [35]. A separate analysis using volumetric RECIST (vRECIST) was also used to differentiate responders (>65 % decrease in tumor volume) from non-responders (<65 % decrease in tumor volume or progression).

### Statistical Analysis

Summary metrics, including the individual lesion volumes, minimum dose to 20 % of the lesion (D_20_), minimum dose to 70 % of the lesion (D_70_), and average dose (D_avg_), between responders and non-responders were assessed using a two-sample *t* test and logistic regression. Results were considered statistically significant at *p* < 0.05. Dose thresholds for assessing response were obtained using receiver operating characteristic (ROC) analysis to determine sensitivity and specificity for response.

## Results

All patients tolerated the imaging procedure without adverse event, and the total time from beginning to end of the PET/MR examination ranged from 42 to 60 min. The fusion of PET and MRI data was accomplished with adequate registration in all cases using rigid registration. The distribution of ^90^Y microspheres was concordant to injection site in all patients (treated lobe:background SUV_mean_ ratios were significantly greater than 1 for all patients (*p* < 0.001). A single case of extrahepatic deposition was identified due to a patent falciform artery. The patient developed no adverse event related to the deposition. No patients had significant toxicity following ^90^Y radioembolization treatment.

### Response Analysis Based on RECIST

**Fig. 1:**
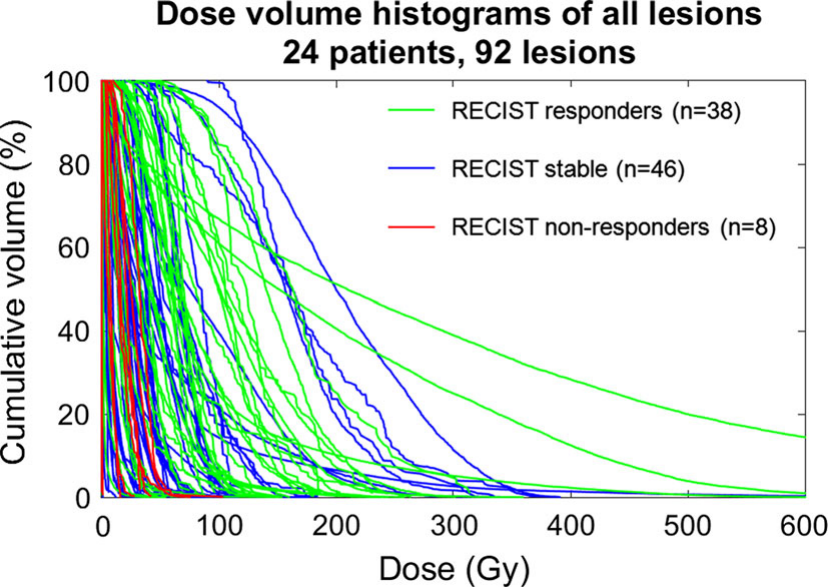
Dose volume histograms of all lesions color-coded by response as defined by RECIST. *Gy* Gray. D_avg_ and D_70_ are significant for predicting response between responding (*green*) and non-responding (*red*) lesions (*p* = 0.0092 and 0.0063, respectively)

**Fig. 2:**
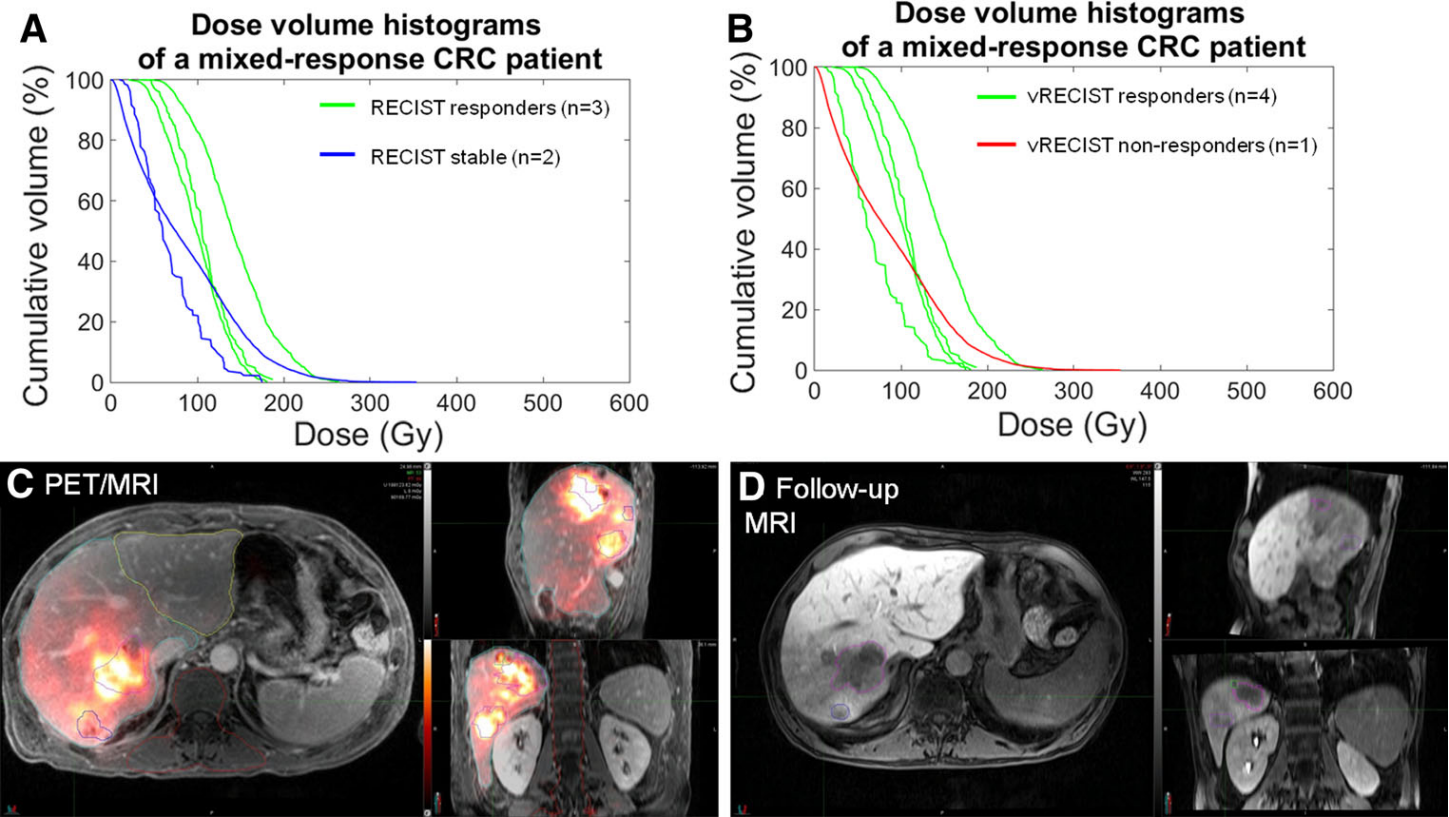
Patient with colorectal carcinoma (CRC) metastases to the liver showing heterogeneous lesion response following lobar treatment. The DVH shows a mix of responders and stable disease, according to RECIST (**A)**, and responders and non-responders, according to vRECIST (**B)**. The PET/MR fused image (**C**) demonstrates the contours of different lesions at baseline as well as the overlay of the ^90^Y microsphere deposition within the treated lobe. Follow-up imaging (**D**) shows the change in lesion size

Using standard RECIST criteria, there were 38 responding lesions, 46 stable lesions, and 8 non-responding lesions across the 24 patients. The relationship of DVH and response is shown in Fig. 1. D_avg_ and D_70_ were statistically significant in predicting response between responders and non-responders (*p* < 0.05, see Table 2). D_avg_ was statistically significant in predicting response between responders and stable lesions (*p* < 0.05, see Table 2); however, D_70_ was not statistically significant for this response pair (*p* > 0.05, see Table 2). No statistical significance was achieved for predicting response between non-responders and stable lesions (*p* > 0.05, see Table 2). In an effort to control for any confounding effects, there was no correlation between response and tumor size (*p* > 0.05). Within individual patients, there was heterogeneous response of lesions to treatment (see Fig. 2A).

**Table 2:** Factors associated with RECIST response on univariate analysis

RECIST *p* values from logistic regression analysis	All lesions		CRC lesions		Hypervascular lesions	
	Average Dose	D70	Average dose	D70	Average dose	D70
Response/progression	0.0092*	0.0063*	0.0452*	>0.05	>0.05	>0.05
Response/stable	0.0291*	>0.05	>0.05	>0.05	>0.05	>0.05
Progression/stable	>0.05	>0.05	>0.05	>0.05	>0.05	>0.05

*Significance achieved at *p* < 0.05

**Fig. 3:**
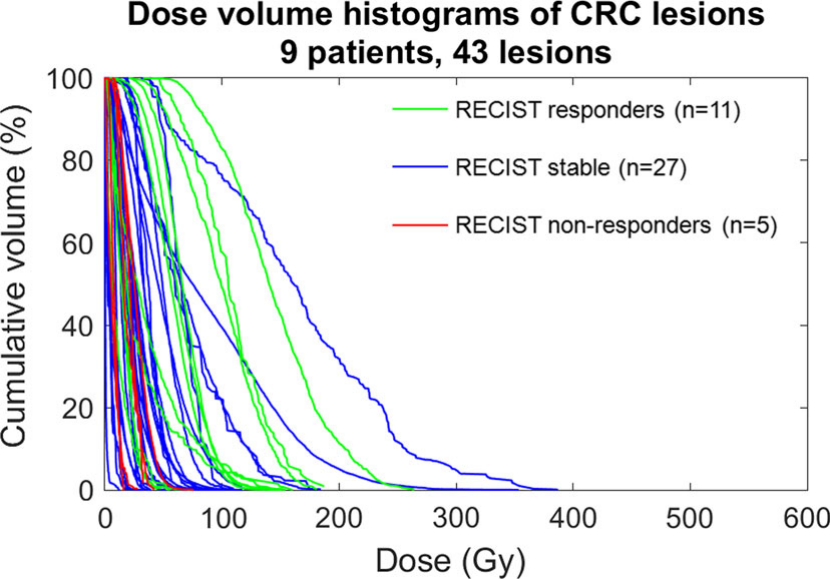
Dose volume histograms of colorectal metastases (CRC) color-coded by response as defined by RECIST. D_avg_ is significant for predicting response between responding (*green*) and non-responding (*red*) lesions (*p* = 0.0452)

Figure 3 shows the relationship of DVH and response for CRC patients (*n* = 9 patients, 43 lesions). D_avg_ between responders and non-responders was the only quantity that achieved statistical significance for predicting response for the CRC lesions (*p* < 0.05, see Table 2).

**Fig. 4:**
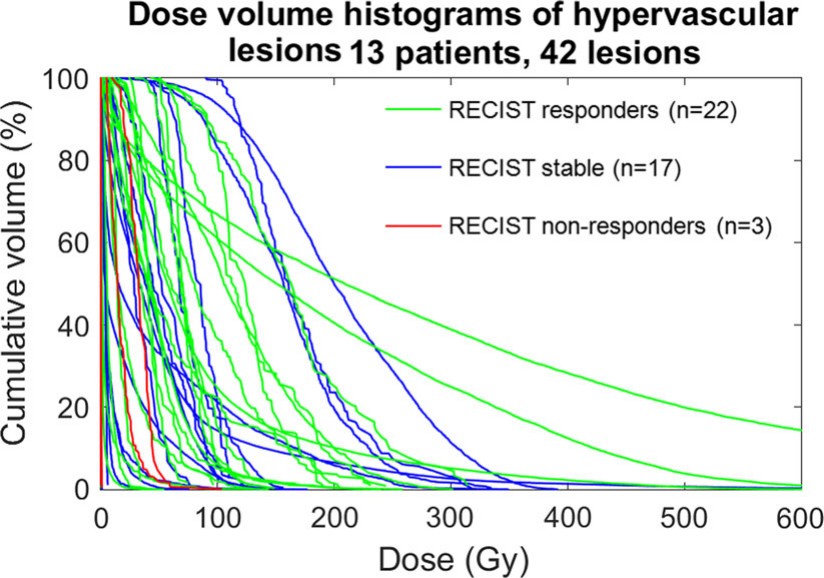
Dose volume histograms of hypervascular lesions (HCC, NET, thymic carcinoid) color-coded by response as defined by RECIST. There were no summary statistics that were significant enough to predict response between any of the response categories (*p* > 0.05, see Table 2)

**Fig. 5:**
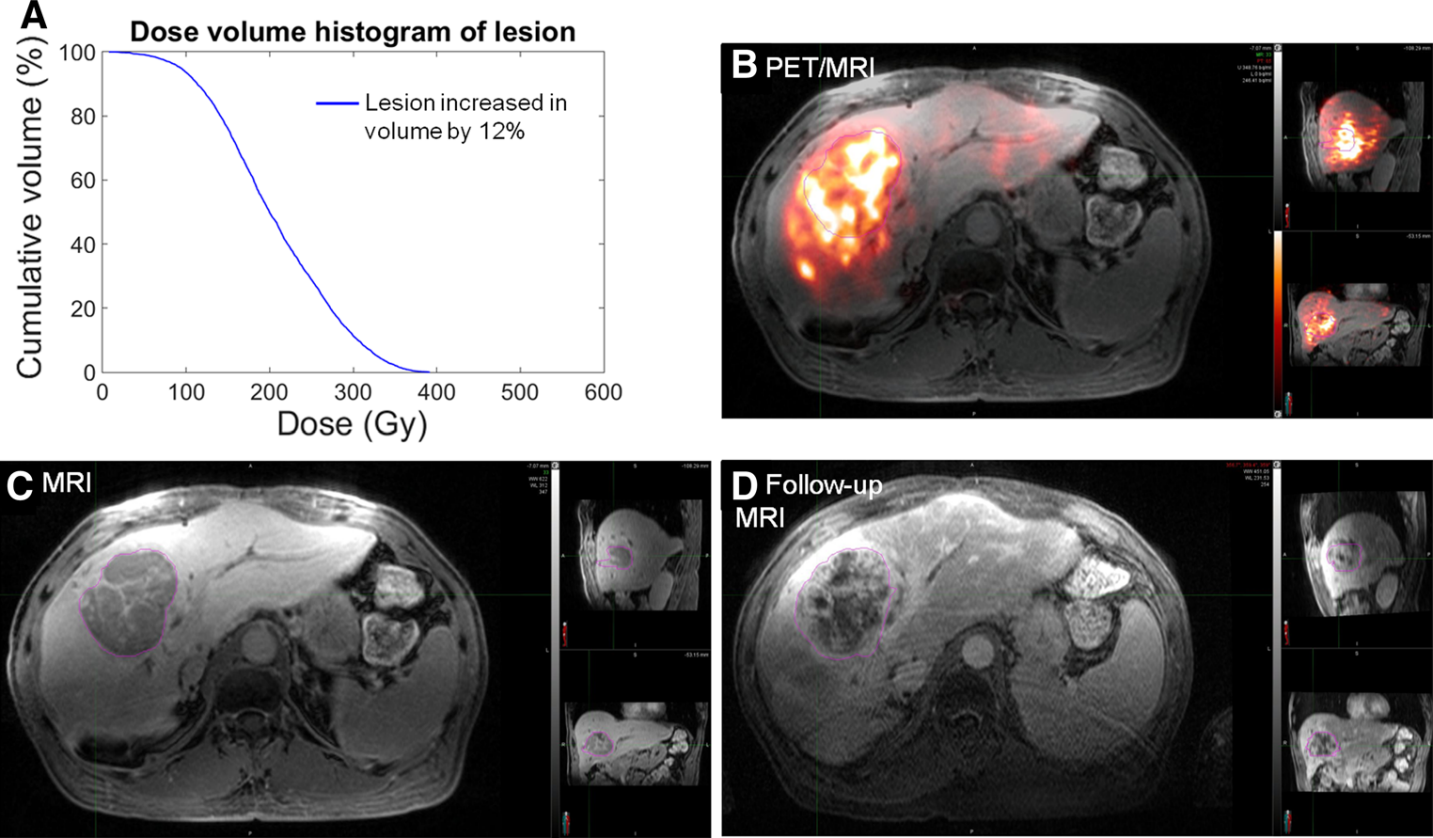
Hepatocellular carcinoma (HCC) lesion representing one of the three outliers among the hypervascular lesions (see Fig. 4). Despite a relatively high delivered dose (**A)**, this lesion did not respond to therapy. PET/MRI (**B)** shows expected deposition. Baseline MRI (**C**) and follow-up MRI (**D)** show stable/no response as defined by RECIST/vRECIST

Figure 4 shows the relationship of DVH and response for hypervascular lesions (HCC, NET, and thymic carcinoid; *n* = 13 patients; 42 lesions). There was no significant relationship between DVH values and response due to the low number (*n* = 3) of non-responding lesions. A single HCC lesion represents one of a few outliers in the data and is shown in Fig. 5 along with the DVH for the lesion. Despite relatively high delivered dose, the lesion did not demonstrate decrease in size and remained primarily enhancing at follow-up imaging acquired 87 days following treatment.

### Response Analysis Based on vRECIST

**Fig. 6:**
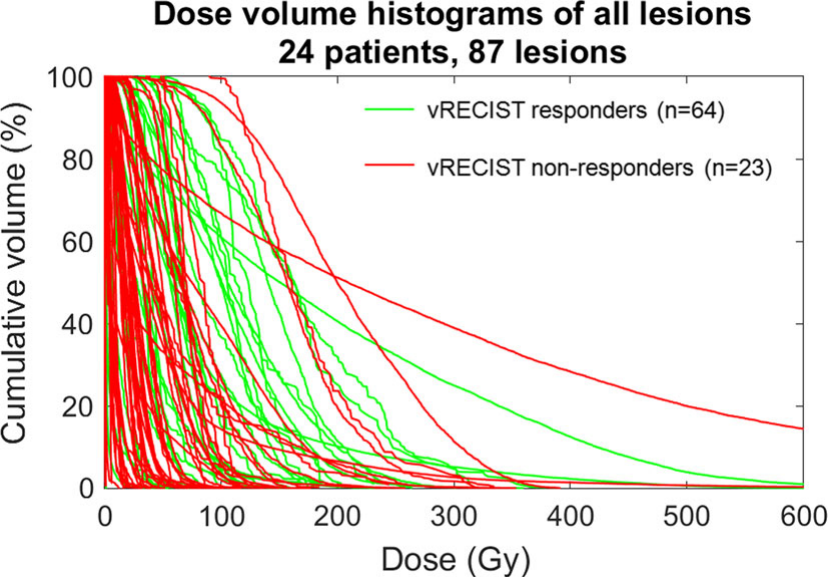
Dose volume histograms of all lesions color-coded by response as defined by vRECIST. D_avg_ and D_70_ are significant for predicting response between responding (*green*) and non-responding (*red*) lesions (*p* = 0.0341 and 0.0194, respectively)

Using vRECIST, there were 64 responding lesions and 23 non-responding lesions across the 24 patients. The relationship of DVH and response is shown in Fig. 6. Both D_avg_ and D_70_ achieved statistical significance in predicting response (*p* < 0.05, see Table 3). Within individual patients, there was heterogeneous response of lesions to treatment (see Fig. 2B).

**Table 3:** Factors associated with vRECIST response on univariate analysis

vRECIST *p* values from logistic regression analysis	All lesions		CRC lesions		Hypervascular lesions	
	Average dose	D70	Average dose	D70	Average dose	D70
Response/progression	0.0341*	0.0194*	0.0004*	0.0004*	>0.05	>0.05

* Significance achieved at *p* < 0.05

**Fig. 7:**
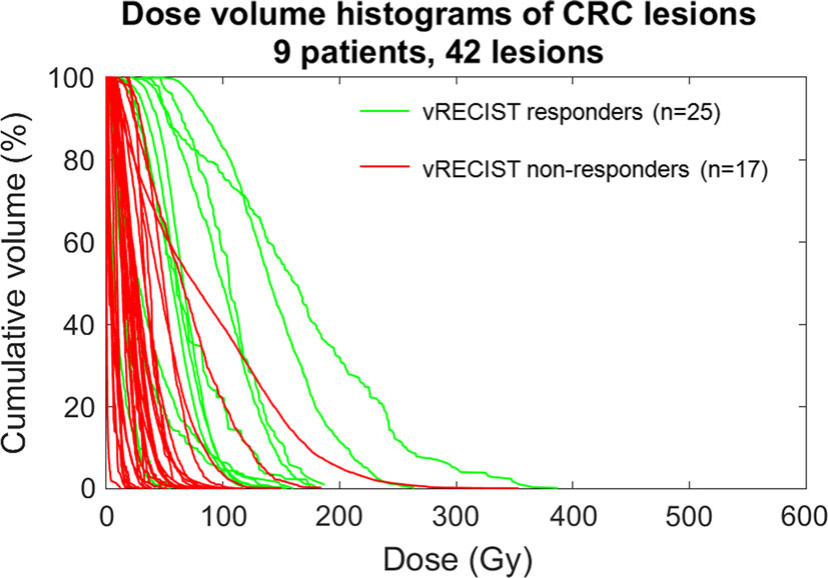
Dose volume histograms of colorectal metastases (CRC) color-coded by response as defined by vRECIST. D_avg_ and D_70_ are significant for predicting response between responding (*green*) and non-responding (*red*) lesions (*p* = 0.0004)

Figure 7 shows the relationship between DVH and response for CRC lesions using vRECIST criteria. Across the 9 patients, there were 25 responding lesions and 17 non-responding lesions. Both D_avg_ and D_70_ achieved statistical significance for predicting response, with equal p values (*p* < 0.05, see Table 3). For CRC lesions, a D_avg_ of 29.8 Gy provided 76.9 % sensitivity and 75.9 % specificity for predicting response; D_70_ of 42.3 Gy provided 61.5 % sensitivity and 96.6 % specificity for predicting response.

**Fig. 8:**
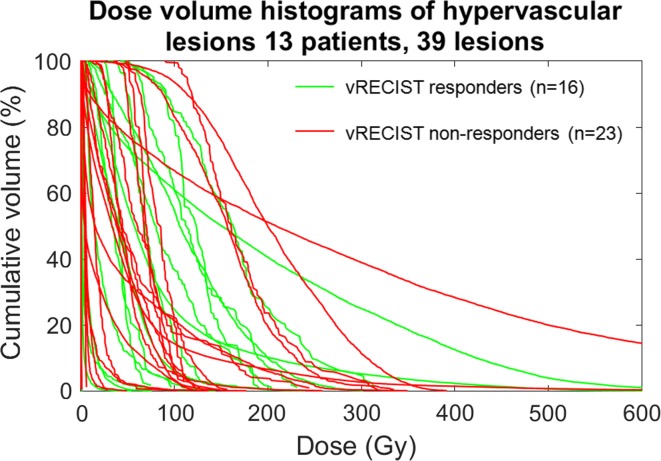
Dose volume histograms of hypervascular lesions (HCC, NET, thymic carcinoid) color-coded by response as defined by vRECIST. There were no summary statistics that were significant enough to predict response between any of the response categories (*p* > 0.05, see Table 3)

Figure 8 shows the relationship between DVH and response for hypervascular lesions using vRECIST criteria. Similar to standard RECIST, these lesions did not achieve statistical significance in predicting response (*p* > 0.05, see Table 3).

## Discussion

There is growing interest in imaging the delivered activity following ^90^Y radioembolization both for confirmation of delivery site and quantification of dose [26, 27, 36]. PET imaging appears to be the most reliable and best option, providing higher spatial resolution and more accurate depiction of uptake than ^90^Y Bremsstrahlung SPECT imaging [26, 38]. In our study, PET/MR imaging of ^90^Y microsphere distribution demonstrated similar quantitative and qualitative results as previously published with PET/CT, including the ability to discern extrahepatic deposition [23, 25, 37].

In our study, the DVH was generated to measure dose distribution within tumors. This method has previously been shown to correlate with tumor response [27]; however, the exact metric D_avg_ or D_70_ remains controversial [40]. When using vRECIST, our results for CRC patients were significant for D_avg_ and D_70_ metrics; however, statistical significance was not achieved for D_70_ when using standard RECIST. Using ROC analysis, we were also able to demonstrate a threshold for vRECIST response in CRC patients at D_avg_ = 29.8 Gy (sensitivity 76.9 %; specificity 75.9 %) and D_70_ = 42.3 Gy (sensitivity 61.5 %; specificity 96.6 %).

Although RECIST is the standard method for assessing lesion response, Tacher et al. recently found that vRECIST was a more accurate predictor of patient survival following transcatheter arterial chemoembolization (TACE) [39]. In our study, we correlated dosimetric quantities with response using both response criteria. Both RECIST and vRECIST resulted in statistically significant results for predicting response across all lesions and for CRC lesions. There was a greater significance achieved using vRECIST as opposed to RECIST for CRC lesions. While vRECIST results were stronger, the average dose was still statistically significant in predicting response between responding and non-responding lesions when using RECIST. Stable disease or disease control, while not the primary goal of therapy, may be a reasonable outcome and was considered as a separate category. Neither vRECIST nor RECIST measurements demonstrated statistical significance in differentiating this category from responders and non-responders.

The inherent value of DVH analysis is that it captures the heterogeneous nature of ^90^Y microsphere deposition. Prior studies have shown wide variations in measured tumor and parenchymal ^90^Y microsphere deposition following lobar administrations [25, 30]. In a recent study, Padia et al. showed heterogeneous ^90^Y microsphere deposition within tumor and portal vein tumor thrombus that appeared to correlate with regions of necrosis on follow-up imaging [25]. Srinivas et al. demonstrated wide variability in dose delivered to 98 HCC lesions [41]. The concept of heterogeneous delivery to the parenchyma and tumors may explain heterogeneous response of different lesions within patients who have large tumor burden, as was seen in our study (Fig. 2-CRC patient). It is possible that distribution of ^90^Y microspheres within the target area is highly dependent on locoregional flow factors, injection rate, proximity and complexity of daughter vessel branching, particle load, and cardiovascular dynamics, in addition to inherent tumor vascularity and necrosis. Most current dosing models assume uniform delivery of activity to the treated region/tumor, which is likely a false assumption. Our study confirms the variable dose distribution and is the first to show significant relationship between the DVH in CRC metastases and response of the lesions on follow-up imaging.

The results of our study represent the first dose–response database generated by PET/MR DVH data for CRC patients undergoing radioembolization treatment. Future adaptive trials may implicate the findings of post-treatment PET/MRI to achieve adequate tumor coverage. Chang et al. published preliminary data suggesting that quantitative PET/CT following ^90^Y radioembolization treatment in HCC could achieve more optimized dose coverage (increase in 40 Gy absorbed dose to tumor) and ultimately a complete response [42].

Our study failed to show a similar significant DVH:response relationship in hypervascular lesions (HCC and NET primarily). In the series published by Srinivas et al., the authors likewise failed to show significant correlation between the mean tumor dose and response in 48 evaluable lesions (21 responders, 27 non-responders) [41]. While their results did not reach significance, there was a trend toward greater response and higher dose. Other authors have demonstrated positive correlation. Kao et al. reported retrospective dose–response information using PET/CT post-treatment DVH analysis, suggesting that complete response could be achieved in HCC patients with a D_70_ > 100 Gy and that this dose level was achieved more easily in smaller tumors (<80 cm^3^) [27]. The lack of significance in our population may be explained by the outlier HCC case and also the small population size. Further research is needed to confirm the positive results shown by others.

There are several limitations of our study. The dose:response data generated represent that acquired on a lesion by lesion basis, which are of great value; however, ultimately patient outcomes and overall survival are better metrics of treatment efficacy. It is our hope that our preliminary results may inform future larger prospective trials with overall survival as the final outcome measure. Another limitation is imperfect registration. While PET/MRI is acquired in a simultaneous manner, improved registration through motion correction algorithms are needed to advance the technological aspects of the study. We were able to achieve satisfactory registration in all cases using MimVista non-deformable registration. Furthermore, in our phantom study and in other phantom studies on PET/CT, recovery for regions 8-37 mm in diameter is only about 50 % for ^90^Y compared to what is recovered when measuring with ^18^F [32, 43]. Even though point spread function (PSF) compensation was included in the reconstruction process, which has been shown to improve contrast recovery and mitigate partial volume effects in PET images [44], counts were still not completely recovered in the reconstructed ^90^Y PET images from ours and other’s phantom studies [33, 41]. Further work with partial volume correction is needed for improving quantitative accuracy, especially for smaller lesions.

Although the results of PET/MR occur after radioembolization, this does not reduce the clinical utility. Immediate predictions (i.e., not waiting for the follow-up imaging study, which usually does not occur for 3 months following therapy) of tumor response could stratify patient therapy based on lesion prognosis. We would hope that this prediction of response could guide further liver directed or systemic therapies, such as cryoablation, microwave ablation, stereotactic radiation, or changes in chemotherapy. Our results provide preliminary data suggesting that PET/MRI and volumetric tumor measurements (vRECIST) may provide a useful metric for predicting response in CRC patients.

In conclusion, simultaneous PET/MR imaging is a feasible way of determining ^90^Y microsphere distribution in the liver. Additional work to improve the quantitative nature of this imaging modality is needed. Future clinical and research applications may yield improvements in radioembolization delivery, dosing, and response assessment.
